# Hierarchical Neurocognitive Model of Externalizing and Internalizing Comorbidity

**DOI:** 10.21203/rs.3.rs-5397195/v1

**Published:** 2025-01-10

**Authors:** Tianye Jia, Chao Xie, Shitong Xiang, Yueyuan Zheng, Chun Shen, Yuzhu Li, Wei Cheng, Nilakshi Vaidya, Zuo Zhang, Lauren Robinson, Jeanne Winterer, Yuning Zhang, Sinead King, Gareth Barker, Arun Bokde, Rüdiger Brühl, Hedi Kebir, Dongtao Wei, Eric Artiges, Marina Bobou, M. Broulidakis, Tobias Banaschewski, Andreas Becker, Christian Buchel, Patricia Conrod, Tahmine Fadai, Herta Flor, Antoine Grigis, Yvonne Grimmer, Hugh Garavan, Penny Gowland, Andreas Heinz, Corinna Insensee, Viola Kappel, Hervé Lemaître, Jean-Luc Martinot, Marie-Laure Martinot, Betteke Noort, Frauke Nees, Dimitri Papadopoulos Orfanos, Jani Penttilä, Luise Poustka, Juliane Frohner, Ulrike Schmidt, Julia Sinclair, Michael Smolka, Maren Struve, Henrik Walter, Robert Whelan, Jiang Qiu, Peng Xie, Barbara Sahakian, Trevor Robbins, Sylvane Desrivières, Gunter Schumann, Jianfeng Feng

**Affiliations:** Fudan University; Institute of Science and Technology for Brain-Inspired Intelligence, Fudan University; Institute of Science and Technology for Brain-Inspired Intelligence, Fudan University; Division of Social Science, Hong Kong University of Science and Technology Fudan Univerisity; Fudan University; Fudan University; Fudan University; Charité Universitätsmedizin Berlin; King’s College London; Oxford; Department of Psychiatry and Psychotherapy CCM, Charité – Universitätsmedizin Berlin, Germany; King’s College London; Department of Psychiatry and Psychotherapy, Technische Universität Dresden, Dresden, Germany; Department of Neuroimaging, King’s College London; Discipline of Psychiatry, School of Medicine and Trinity College Institute of Neuroscience, Trinity College Dublin, Dublin, Ireland; Physikalisch-Technische Bundesanstalt; Centre for Population Neuroscience and Stratified Medicine (PONS), Department of Psychiatry and Neuroscience, Charité Universitätsmedizin Berlin, Germany; Key Laboratory of Cognition and Personality (SWU), Ministry of Education, Chongqing, China; INSERM/Université Paris-Saclay; Social Genetic and Developmental Psychiatry Centre, Institute of Psychiatry, Psychology and Neuroscience, King’s College London, London, SE5 8AF, UK; Department of Psychiatry, University of Southampton, United Kingdom;; Central Institute of Mental Health, Mannheim; Department of Child and Adolescent Psychiatry and Psychotherapy, University Medical Center Göttingen, Göttingen, Germany; University Medical Center Hamburg-Eppendorf; University of Montreal, Canada; University Medical Centre Hamburg-Eppendorf, House W34, 3.OG, Hamburg, Germany; Central Institute of Mental Health Medical Faculty Mannheim Heidelberg University; Department of Child and Adolescent Psychiatry and Psychotherapy, Central Institute of Mental Health, Medical Faculty Mannheim, Heidelberg University, Mannheim, Germany; University of Vermont; University of Nottingham; Charité Universitätsmedizin, Berlin; Georg-Elias-Müller-Institute of Psychology, Department of Clinical Psychology and Psychotherapy, University of Göttingen, Gosslerstraße 14, 37073 Göttingen, Germany; Department of Cognitive and Clinical Neuroscience, Central Institute of Mental Health, Mannheim, Germany; Groupe d’Imagerie Neurofonctionnelle; Institut National de la Santé et de la Recherche Médicale; Institut National de la Santé et de la Recherche Médicale, INSERM U 1299 “Trajectoires développementales & psychiatrie”, University Paris-Saclay, CNRS; Department of Psychology, MSB Medical School Berlin, Rüdesheimer Str. 50, 14197 Berlin, Germany; University Medical Center Schleswig-Holstei, n Kiel University; Department of Social and Health Care, Psychosocial Services Adolescent Outpatient Clinic Kauppakatu 14, Lahti, Finland; Department of Child and Adolescent Psychiatry and Psychotherapy, University Medical Centre Gottingen; Department of Psychiatry and Psychotherapy, Technische Universitat Dresden, Dresden, Germany; King’s College London; University of Southampton; Technische Universität Dresden; Department of Cognitive and Clinical Neuroscience, Central Institute of Mental Health, Mannheim, Germany; Department of Psychiatry and Psychotherapy CCM, Charite - Universitatsmedizin Berlin, corporate member of Freie Universitat Berlin, Humboldt-Universitat zu Berlin, and Berlin Institute of Health, Be; School of Psychology and Global Brain Health Institute, Trinity College Dublin, Ireland; Southwest University; The First Affiliated Hospital of Chongqing Medical University; University of Cambridge; University of Cambridge; Institute of Psychiatry, Psychology & Neuroscience, King’s College London, United Kingdom; Charite Universitaetsmedizin Berlin; Fudan University

## Abstract

Mounting evidence suggests hierarchical psychopathology factors underlying psychiatric comorbidity. However, the exact neurobiological characterizations of these multilevel factors remain elusive. In this study, leveraging the brain-behavior predictive framework with a 10-year longitudinal imaging-genetic cohort (IMAGEN, ages 14, 19 and 23, *N* = 1,750), we constructed two neural factors underlying externalizing and internalizing symptoms, which were reproducible across six clinical and population-based datasets (ABCD, STRATIFY/ESTRA, ABIDE II, ADHD-200 and XiNan, from age 10 to age 36, *N* = 3,765). These two neural factors exhibit distinct neural configurations: hyperconnectivity in impulsivity-related circuits for the externalizing symptoms and hypoconnectivity in goal-directed circuits for the internalizing symptoms. Both factors also differ in their cognitive-behavior relevance, genetic substrates and developmental profiles. Together with previous studies, these findings propose a hierarchical neurocognitive spectral model of comorbid mental illnesses from preadolescence to adulthood: a general neuropsychopathological (NP) factor (manifested as inefficient executive control) and two stratified factors for externalizing (deficient inhibition control) and internalizing (impaired goal-directed function) symptoms, respectively. These holistic insights are crucial for the development of stratified therapeutic interventions for mental disorders.

## Introduction

Psychiatric comorbidity is prevalent and often leads to more severe prognoses^1^, posing a major challenge to the current mental health diagnostic system ^2^. In response, the Hierarchical Taxonomy of Psychopathology (HiTOP) was proposed to categorize the complex psychiatric comorbidities into a general factor alongside multiple stratified transdiagnostic spectra, for instance, the externalizing (aggressive and hyperactive-impulsive) vs. internalizing (anxious and depressive) spectrum ^3,4^. Recently, our research team identified a prefrontal-related general Neuropsychopathological (NP) factor underlying both externalizing and internalizing symptoms from preadolescence to early adulthood ^5^. However, the neurobiological mechanisms of externalizing and internalizing disorders and the interaction of general-stratified factors during development remain elusive.

Another dimensional framework, the Research Domain Criteria (RDoC) ^6^, was developed to advance the investigation of neurobiological foundations of dimensional psychopathology. However, the research progress in uncovering the stratified neural bases of externalizing and internalizing disorders has remained slow^7^. This sluggishness is partly due to the historical emphasis on localizing brain abnormalities at the regional level in psychiatric disorders ^8–10^. It is crucial to recognize that different brain regions do not function or develop independently; instead, they work in distributed and anatomically interconnected systems^11,12^. The above evidence hence suggests that distinct regionalized brain markers of psychiatric disorders might be located within a common psychopathological brain network. This hypothesis has recently gained support from normative network mapping and connectivity-based transdiagnostic studies^13,14^, emphasizing the importance of network-based approaches in unifying region-level heterogeneous neural underpinnings of psychiatric disorders ^15,16^.

Furthermore, previous transdiagnostic neuroimaging studies have predominantly employed a cross-sectional approach^17–19^, thereby overlooking the developmental perspective on how the general and stratified neural substrates manifest and evolve longitudinally^3^, especially during critical developmental periods like adolescence. Utilizing a longitudinal large-scale imaging dataset, we can further elucidate the nuanced interplay between the enduring and phasic neural mechanisms of psychiatric comorbidity^20^. This approach will significantly advance our understanding of the onset and progression of psychiatric comorbidity.

The present study addresses three major questions regarding the specific transdiagnostic neural bases of externalizing and internalizing symptoms: (1) Can we identify stratified cross-disorder neural factors for externalizing and internalizing symptoms, respectively? (2) Do the two stratified neural factors exhibit distinct characterisations regarding neurobiological risk factors and clinical conditions? (3) How can we synthesize the general and stratified neural factors into a hierarchical neurocognitive model of comorbid psychopathology?

## Result

### Stratified neural factors of externalizing and internalizing symptoms

**Figure 1:**
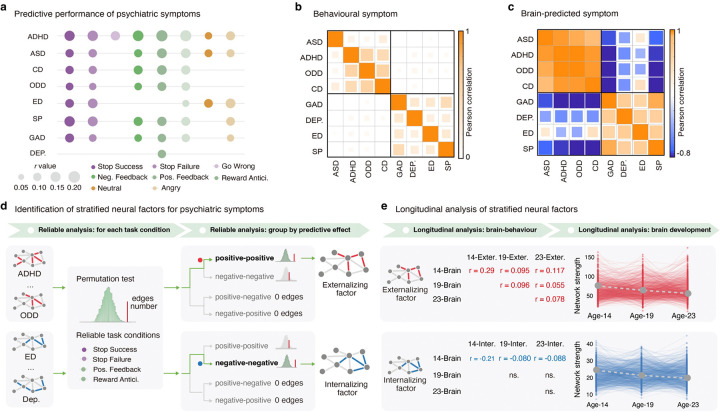
Identification of the stratified neural factors. **a.** The predictive performance of behavioral symptoms related to psychiatric symptoms with the task-based connectivity model. Task-based connectivity was estimated from the EFT (angry and neutral conditions), the MID task (reward anticipation, positive reward feedback and negative reward feedback conditions) and the SST (go-wrong, stop-success and stop-failure conditions). **b**. The correlation matrix of the behavioral symptoms. The externalizing and internalizing symptoms showed high intra-correlations within their respective psychiatric domain, but low correlations between each other. Externalizing symptoms consisted of ASD, ADHD, CD and ODD. Internalizing symptoms comprised GAD, Dep., ED and SP. **c**. The correlation matrix of the brain-predicted symptoms. **d**. With a two-step reliable analysis, we identified two stratified neural factors for externalizing and internalizing symptoms, respectively. We first identified task conditions with reliable stratified cross-disorder edges, which are defined as predictive edges that only predict externalizing but not internalizing symptoms and vice versa. We found that only conditions from the SST and MID task had significantly more stratified cross-disorder edges than a random observation. Then, we further identified which type of cross-disorder edges reliably predict externalizing or internalizing symptoms, which was termed the stratified factors. We discovered that the externalizing neural factor consisted of positive-positive cross-disorder edges (positively predicted at least two externalizing symptoms), while the internalizing neural factor comprised negative-negative cross-disorder edges (negatively predicted at least two internalizing symptoms). **e**. We checked the longitudinal predictive effects and developmental trajectories of the stratified factors across ages 14, 19 and 23. EFT, emotional face task; MID, monetary incentive delay task; SST, stop signal task; ADHD, attention-deficit/hyperactivity disorder; ASD, autism spectrum disorder; CD, conduct disorder; ODD, oppositional defiant disorder; GAD, general anxiety disorder; Dep., depression; ED, eating disorder; SP, specific phobia; 14-brain, brain at age 14; 19-brain, brain at age 19; 23-brain, brain at age 23; 14-exter., externalizing symptoms at age 14; 19-exter., externalizing symptoms at age 19; 23-exter., externalizing symptoms at age 23; 14-inter., internalizing symptoms at age 14; 19-inter., internalizing symptoms at age 19; 23-inter., internalizing symptoms at age 23;

Our previous study found significant predictive effects of task-based connectomes on eight psychiatric symptoms in 14-year-old participants from the IMAGEN study (Fig. 1a and **Supplementary Tables 1 and 2**, N = 1,750) ^5^. For each psychiatric symptom, we generated a brain-predicted measure. Interestingly, we observed that there were significantly higher similarities between brain-predicted symptoms than the observed psychiatric symptoms (externalizing symptoms: brain-predicted *r*_mean_ = 0.91, observed *r*_mean_ = 0.37, *P*_perm_ < 0.001 for the difference; internalizing symptoms: brain-predicted *r*_mean_ = 0.52, observed *r*_mean_ = 0.28, *P*_perm_ < 0.001 for the difference) (Fig. 1b and c). The results thus suggested substantial shared neural bases within externalizing and internalizing symptoms.

We next aimed to identify specific neural factors, termed as ‘stratified neural factors’, comprising cross-disorder edges that predicted two or more symptoms from a single psychiatric domain (externalizing or internalizing), while not predicting any symptoms from the other domain (Fig. 1d). We found that stratified cross-disorder edges were consistently and reliably identified from task conditions relating to inhibitory control and reward sensitivity (i.e. stop success, stop failure, positive reward feedback and reward anticipation; all *P*_perm_ < 0.01, **Supplementary Table 3**).

We further ascertained the neural mechanisms of the stratified cross-disorder edges in terms of their predictive effects (Fig. 1d). Intriguingly, we observed a double dissociation effect: externalizing symptoms were reliably associated with positive-positive cross-disorder edges (*n*_*edge*_ = 1,268, *P*_*perm*_ < 0.001, showing positive correlations with externalizing symptoms), whereas internalizing symptoms were reliably associated with negative-negative cross-disorder edges (*n*_*edge*_ = 469, *P*_*perm*_ < 0.001, showing negative correlations with internalizing symptoms) (**Supplementary Table 4**). Therefore, in the following analyses, the summed functional connectivity (FC) strength of positive-positive cross-disorder edges will be referred to as the externalizing neural factor, and the summed strength of negative-negative cross-disorder edges as the internalizing neural factor.

### Longitudinal analysis of stratified neural factors

As previous research has highlighted the stability of internalizing and externalizing behaviors over time ^21^, we next examined the longitudinal predictive performance of the two stratified neural factors from adolescence to early adulthood over a 10-year span (Fig. 1e). The externalizing factor demonstrated consistent performance for longitudinal prediction across ages 14, 19 and 23. To elaborate, the externalizing neural factor estimated at age 14 could significantly predict externalizing symptoms at ages 19 (N = 1,045, *r* = 0.095, *t* = 3.09, *P*_*one–tailed*_ = 0.001, **Supplementary Table 5A**) and 23 (N = 1,043, *r* = 0.117, *t* = 3.79, *P*_*one–tailed*_ = 7.98E-05, **Supplementary Table 5A**). Also, the externalizing neural factors estimated at ages 19 and 23 could predict their corresponding externalizing symptoms at the same age or later age (brain and symptoms both at age 19, N = 1,095, *r* = 0.096, *t* = 3.20, *P*_*one–tailed*_ =7.05E-04; brain at age 19 and symptoms at age 23, N = 1,029, *r* = 0.055, *t* = 1.77, *P*_*one–tailed*_ =0.038; brain and symptoms both at age 23, N = 937, *r* = 0.078, *t* = 2.388, *P*_*one–tailed*_ =0.009, **Supplementary Table 5C and 5E**). In contrast, only the internalizing neural factor estimated at age 14 could significantly predict future internalizing symptoms measured at ages 19 and 23 (age 19, N = 1,045, *r* = −0.080, *t* = −2.59, *P*_*one–tailed*_ = 0.005; age 23, N = 1,043, *r* = −0.088, *t* = −2.84, *P*_*one–tailed*_ = 0.002, **Supplementary Table 5B**), but not for the internalizing neural factors estimates at ages 19 and 23 (**Supplementary Table 5D and 5F**).

We next examined the longitudinal changes of the two stratified neural factors across ages 14, 19 and 23 (Fig. 1e). While both the externalizing and internalizing neural factors maintained highly significant positive FC strengths throughout ages 14, 19 and 23 (All *t* > 100, Cohen ‘*d* > 3.1, P < 0.001), steady decreases in FC strength from age 14 to age 23 were also observed for both neural factors (externalizing slop *β*_*mean*_ = −9.44, *t* = −17.64, Cohen’s *d* = −0.68, *P*_*two–tailed*_ = 1.95E-57; internalizing slop *β*_*mean*_ = −2.33, t = − 18.89, Cohen’s *d* = −0.73, *P*_*two–tailed*_ = 4.83E-64). During this critical developmental period, the normative decrease in connectivity strength could be explained by the neural pruning for a more efficient brain information process^22^. Lastly, we investigated the associations between psychiatric symptoms at age 14 and the rate of decline in the stratified neural factors from age 14 to 23. We observed that individuals with higher baseline externalizing symptoms may have undergone an under-pruning process of the externalizing neural factor from adolescence to early adulthood (N = 575, *r* = −0.20, *t* = −4.92, *P*_*two–tailed*_ = 1.12E-06); conversely, individuals with higher baseline internalizing symptoms experienced an over-pruning process of the internalizing neural factor from adolescence to early adulthood (N = 575, *r* = 0.12, *t* = 2.96, *P*_*two–tailed*_ = 0.003). These results indicated that while both the externalizing and internalizing behavioral domains showed strong within-domain intra-correlations for both observed and neural predicted symptoms, each behavior domain may be represented by a distinct cross-disorder neural substrate.

### Neuroanatomical characterization of stratified neural factors

**Figure 2:**
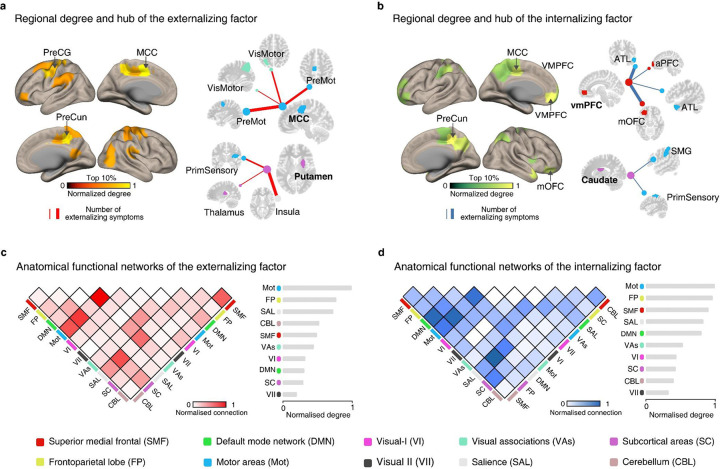
Multilevel neuroanatomical characterization of the two stratified neural factors. a and b. The top 10% nodes and hub node connections (with high regional connections) in the externalizing and internalizing factor. The color bar indicates the normalized node degree (that is, the number of connections with other nodes). c. and d. The functional connections of the externalizing and internalizing factors shared similar large-scale network configurations that both were mainly localized between the motor, frontoparietal and salience networks. The color bar indicates the strength of normalized inter- or intra-network connections, where the number of connections between or within networks was divided by the largest connection number observed.

We characterized the above two stratified neural factors at multiple neuroanatomical levels. In terms of the regional network degree, the externalizing neural factor was mainly located in brain regions such as the middle cingulate cortex (MCC), precentral gyrus (PreCG), precuneus (PreCun), supramarginal gyrus (SMG), and putamen (Fig. 2a and **Supplementary table 6a**), which were commonly implicated in the habitual control process ^23^. In contrast, the internalizing neural factor was primarily enriched in regions such as the precuneus (PreCun), ventromedial prefrontal cortex (vmPFC), medial orbitofrontal cortex (mOFC), and caudate (Fig. 2b and **Supplementary table 6b**), all known to play crucial roles in goal-directed processing ^24^.

Next, while the two stratified neural factors did not share overlapping edges by definition, they did share similar network configurations at a higher neuroanatomical level. For instance, the externalizing and internalizing neural factors exhibited similar network-level configurations, primarily in the motor, frontoparietal, and salience networks (Fig. 2c, d and **Supplementary table 7**). Notably, the salience network plays a pivotal role in attending to motivational stimuli and recruiting appropriate functional brain-behavior networks to modulate behavior ^25^. Therefore, the hyperconnectivity of the externalizing neural factor (e.g. in high-risk individuals) might be associated with excessive perception of external stimuli and a lack of inhibitory control over automatic responses ^26,27^. Conversely, the hypoconnectivity of the internalizing neural factor might be related to limited salience processing, resulting in difficulties in engaging goal-directed behaviors in individuals with internalizing disorders ^28^.

### Functional and genetic bases of stratified neural factors

We then investigated the associations between task performance measures with the two stratified neural factors. Both the externalizing and internalizing neural factors showed significant negative correlations with accuracies in the monetary incentive delay (MID) task (externalizing: N = 1,620, *r* = −0.14, *t* = −5.73, *P*_*two–tailed*_ = 1.20E-08; internalizing: N = 1,620, *r* = −0.08, *t* = −3.22, *P*_*two–tailed*_ = 0.001) and the go-trials in the stop-signal task (SST) (externalizing: N = 1,567, *r* = −0.26, *t* = −10.62, *P*_*two–tailed*_ = 1.81E-25; internalizing: N = 1,567, *r* = −0.20, *t* = −8.01, *P*_*two–tailed*_ = 2.30E-15), but no significant associations with the reaction time in the MID task (externalizing: N = 1,620, *r* = −0.05, *t* = −1.97, *P*_*two–tailed*_ = 0.05; internalizing: N = 1,620, *r* = −0.02, *t* = −0.84, *P*_*two–tailed*_ = 0.40) nor stop signal delay in the SST (externalizing: N = 1,567, *r* = −0.04, *t* = −1.51, *P*_*two–tailed*_ = 0.13; internalizing: N = 1,567, *r* = −0.04, *t* = −1.77, *P*_*two–tailed*_ = 0.08). These results were similar to the general neural factor (i.e. the *NP*-factor) findings in our previous study ^5^.

**Figure 3:**
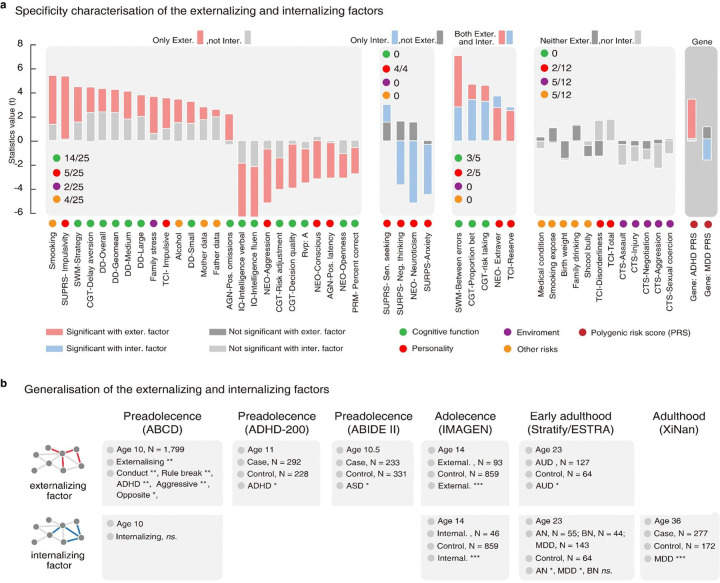
Functional specificity and generalization of the stratified neural factors. **a**. The externalizing factor was specifically associated with most cognitive functions (14 of 25). The internalizing factor was specifically correlated with personality traits (5/5), especially neuroticism and anxiety. Two executive function measurements (between errors in SWM and proportion bet in CGT) and two personality traits (extroversion of NEO and reserve of TCI) showed distinct associations with externalizing and internalizing symptoms. The polygenic risk score (PRS) of ADHD and MDD showed a specific association with externalizing and internalizing factors, respectively. **b** Generalization of the *NP* factor across multiple developmental periods from preadolescence to adulthood in both population and clinical case-control datasets (ABCD, N = 1799; ADHD-200, N = 520; ABIDE II, N = 564; IMAGEN N = 998; STRATIFY and ESTRA, N = 433; and XiNan, N = 449). The significance level (that is, the grey color) was given as a false discovery rate (fdr) of 0.05. The *P* values were reported as the original value and could survive the multiple testing correction with Benjamin–Hochberg procedure. AGN, Affective Go-No Go; BMI, body mass index; DD, Delay Discounting Task, which measured ‘waiting’ impulsivity^47^; MidOcci, middle occipital cortex; MidPFC, middle prefrontal cortex; NEO, NEO Personality Inventory; RVP: A, Target Sensitivity from Rapid Visual Information Processing task; PRM, Pattern Recognition Memory task; SURPS, Substance Use Risk Personality Scale; SWM, Spatial Working Memory task; TCI, Temperament and Character Inventory–Revised. AN, anorexia nervosa; BN, bulimia nervosa; AUD, alcohol use disorder; MDD, major depressive disorder;

Next, we examined the functional specificity of the two stratified neural factors across a wide range of cognitive-behavioral phenotypes. The externalizing neural factor exhibited specific associations with impulsive and substance use behaviors (Fig. 3a, **Supplementary Table 8a**), where impulsivity is a characteristic feature of externalizing disorders and a known risk factor for future substance abuse ^29^. In contrast, the internalizing neural factor was primarily correlated with maladaptive traits, such as neuroticism and negative thinking (Fig. 3a, **Supplementary Table 8b**), where neuroticism plays a pivotal role in longitudinally predicting various internalizing disorders, like anxiety and depression ^30^.

At last, we investigated whether the two stratified neural factors had different genetic substrates by examining their associations with the polygenic risk scores (PRS) of attention-deficit/hyperactivity disorder (ADHD) and major depressive disorder (MDD) as the representations of externalizing and internalizing disorders, respectively. We observed a significant association of the externalizing neural factor with an increased PRS of ADHD (N = 1,594) (*r* = 0.082, *t* = 3.27, *P*_*one–tailed*_ = 5.31E-05), but not with the PRS of MDD (*r* = 0.024, *t* = 0.96, *P*_*two–tailed*_ = 0.33. Conversely, a lower internalizing neural factor was only correlated with the PRS of MDD (N = 1,594) (*r* = −0.04, *t* = −1.74, *P*_*one–tailed*_ = 0.041), but not with that of ADHD (*r* = 0.008, *t* = 0.32, *P*_*two–tailed*_ = 0.74). The above results hence suggested that the externalizing and internalizing neural factors have distinct behavioral and genetic implications for their corresponding psychiatric comorbidity.

### Generalization of stratified neural factors

We evaluated the generalization performance of the two stratified neural factors in multiple population and clinical datasets.

First, with the MID task and SST in the Adolescent Brain Cognitive Development cohort (ABCD) dataset^31^, we found that the externalizing neural factor was significantly correlated with a wide range of externalizing symptoms at age 10 (N = 1,799), including externalizing (*r* = 0.059, *t* = 2.51, *P*_*one–tailed*_ = 0.006), rule break (*r* = 0.070, *t* = 2.97, *P*_*one–tailed*_ = 0.002), conduct (*r* = 0.063, *t* = 2.69, *P*_*one–tailed*_ = 0.006), aggressive (*r* = 0.057, *t* = 2.41, *P*_*one–tailed*_ = 0.008), oppositional defiant (*r* = 0.054, *t* = 2.30, *P*_*one–tailed*_ = 0.011), and attention symptoms (*r* = 0.050, *t* = 2.10, *P*_*one–tailed*_ = 0.018). In addition, the externalizing neural factor estimated at age 10 could also predict the summary score of all externalizing symptoms at age 11 (N = 1,042, *r* = 0.052, *t* = 1.67, *P*_*one–tailed*_ = 0.047), as well as the subscores (ADHD: *r* = 0.078, *t* = 2.49, *P*_*one–tailed*_ = 0.007; opposite: *r* = 0.062, *t* = 2.03, *P*_*one–tailed*_ = 0.021; rule break: *r* = 0.062, *t* = 2.00, *P*_*one–tailed*_ = 0.022). In contrast, the internalizing neural factor showed no significant association with the internalizing-related symptom at ages 10 or 11.

Next, we compared diagnosed patients (marked as severe or high risk for at least one externalizing or internalizing disorder) with healthy controls for the two stratified neural factors in the IMAGEN and ABCD datasets. For the externalizing neural factor, we found that externalizing participants had significantly higher factor scores than control groups in both IMAGEN (externalizing participants N = 93, controls N = 859, t = 7.10, Cohen’s *d* = 0.78, *P*_*one–tailed*_ = 1.24e-12) and ABCD datasets (externalizing participants N = 206, controls N = 1,596, t = 2.67, Cohen’s *d* = 0.20, *P*_*one–tailed*_ = 0.003). However, the comparison of the internalizing neural factor between diagnosed patients and healthy controls was only significant in IMAGEN (internalizing participants: N = 46, controls N = 859, t = −3.42, Cohen’s *d* = 0.52, *P*_*one–tailed*_ = 3.29E-04), but not in ABCD dataset (internalizing participants N = 32, controls N = 1,596, t = 1.85, Cohen’s *d* = 0.33, *P*_*one–tailed*_ = 0.97).

Further, we investigated whether the two stratified neural factors had clinical relevance in the case-control STRATIFY and ESTRA cohort (age = 23) with the SST^32^. We found that the externalizing and internalizing neural factors were differentially associated with psychiatric disorders. To elaborate, the externalizing neural factor of alcohol use disorder (AUD) patients (N = 127) was significantly higher than in the healthy controls (N = 64) (t = 1.82, Cohen’s *d* = 0.28, *P*_*one–tailed*_ = 0.035), but no significant group differences were found for this neural factor between healthy controls (N = 64) and patients with internalizing disorders (Anorexia Nervosa: N = 55, t = −0.54, Cohen’s *d* = 0.10, *P*_*two–tailed*_ = 0.059; Bulimia Nervosa: N = 44, t = 1.47, Cohen’s *d* = 0.29, *P*_*two–tailed*_ = 0.15; Major Depression: N = 143, t = 1.44, Cohen’s *d* = 0.22, *P*_*two–tailed*_ = 0.15). On the other hand, the internalizing neural factor was significantly lower in patients with internalizing disorders than in healthy controls (N = 64) (internalizing patients: N = 242, t = −2.31, Cohen’s *d* = 0.32, *P*_*one–tailed*_ = 0.011; Anorexia Nervosa: N = 55, t = −3.04, Cohen’s *d* = 0.56, *P*_*one–tailed*_ = 0.002; Bulimia Nervosa: N = 44, t = −0.83, Cohen’s *d* = 0.16, *P*_*one–tailed*_ = 0.20; Major Depression: N = 143, t = −2.04, Cohen’s *d* = 0.31, *P*_*one–tailed*_ = 0.021), but no significant group difference was found for the alcohol use disorder (N = 127, t = −0.92, Cohen’s *d* = 0.14, *P*_*two–tailed*_ = 0.36).

Finally, we found that the externalizing factor generated using resting-state fMRI (with the same set of FC as defined in the IMAGEN cohort) was significantly higher in the ASD patients (N = 233) than in healthy controls (N = 331) (ABIDEII, Mean age = 10.5, *t* = 1.90, Cohen’s *d* = 0.08, *P*_*one–tailed*_ = 0.01). A significant difference were also obersved in the group comparison between ADHD patients (N = 292) and healthy controls (N = 228) (ADHD-200, Mean age = 11, t = 2.06, Cohen’s *d* = 0.18, *P*_*one–tailed*_ = 0.020). Furthermore, the internalizing factor generated using resting-state fMRI (with the same set of FC as defined in the IMAGEN cohort) of depressive patients (N = 277) was significantly lower than the control group (N = 172) (XiNan dataset, Mean age = 36.1 *t* = −3.11, Cohen’s *d* = −0.30, *P*_*one–tailed*_ = 9.67e-04). These results further supported the distinct contribution of externalizing and internalizing neural factors to externalizing and internalizing comorbidity, respectively.

### Neural specificities of the three cross-disorder networks

**Figure 4:**
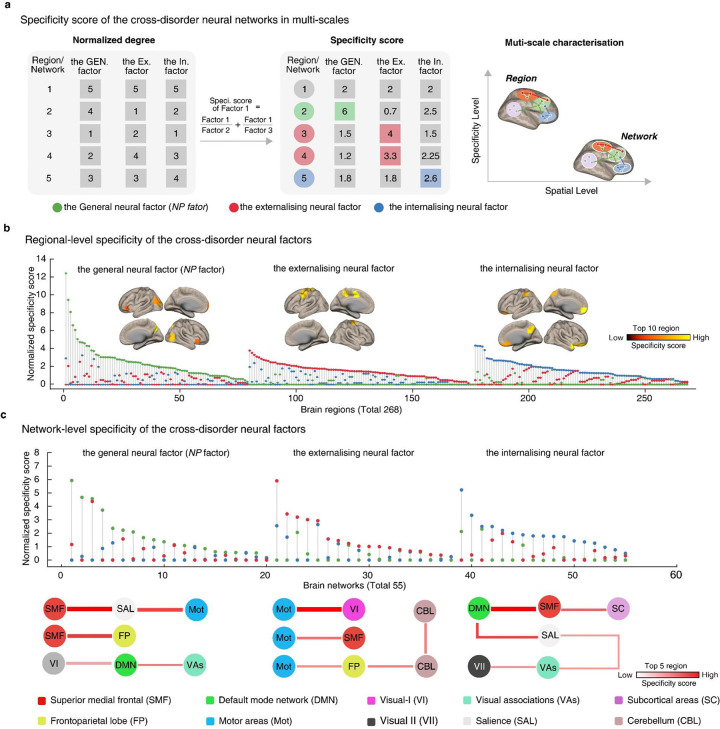
Characterizing specificity of the cross-disorder neural factors at the levels of brain regions and extended networks. **a.** Specificity score comparison of the three cross-disorder networks using regional-level node degree as an example. We first normalized the node degree to facilitate subsequent cross-factor comparisons. Then, we used a weighted method for calculating the regional specificity score with the formula: specificity score of Factor 1 = Factor 1/Factor 2 + Factor 1/Factor 3. By weighting the contribution of the brain region in the other two cross-disorder factors (Factor 2 and Factor 3), the estimated specificity score provides a more robust measure of the unique contribution of each brain region within this cross-disorder factor (Factor 1). Building upon previous findings ^13^, we hypothesize that as the brain scale increases from region to network, the specificity between cross-disorder factors will decrease. **b and c**. For each brain region and network, specificity scores were estimated for all three cross-disorder factors. The maximum specificity score for each ROI is considered indicative of its specificity to that cross-disorder factor. Then, we estimated a specificity distance, which is computed by subtracting the minimum specificity score from the maximum specificity score. This distance is interpreted as the uniqueness of this cross-disorder factor compared to others.

Our previous study identified a general neural factor (*NP* factor) across the externalizing and internalizing symptoms. Here, we would like to closely examine the specific configurations of the three cross-disorder neural factors (one general and two stratified neural factors) based on the Specificity Score, i.e., the contribution of a factor after controlling the other two factors (Fig. 4a, with further details available in the Method).

We first estimated the Specificity Score of each brain region for the three cross-disorder neural factors respectively (Fig. 4b, total 268 regions). We found that 79 regions exhibited predominant associations (i.e. with the highest Specificity Score) with the general *NP* factor, with the most prominent regions including the ventral precuneus, middle occipital cortex, and inferior frontal cortex (**Supplementary Table S9 A**). Additionally, 97 regions were associated predominantly with the externalizing neural factor, most notably the primary sensorimotor cortex areas such as the precentral and middle cingulate cortex (**Supplementary Table S9B**). Finally, 92 regions showed predominant associations with the internalizing neural factor, with notable regions including the medial prefrontal cortex and orbitofrontal cortex (**Supplementary Table S9C**). The Specificity Scores of the three cross-disorder neural factors were significantly different (*F*_(2,265)_ = 15.80, *P* = 3.31E-07), with the general *NP* factor demonstrating significantly higher scores compared to either the externalizing or internalizing counterparts (*NP* vs externalizing: *t*_(174)_ = 4.70, *P* = 5.17E-06; *NP* vs internalizing: *t*_(169)_ = 3.85, *P* = 1.63E-04).

As previous findings have suggested that heterogeneous regions of the same psychiatric disorder might be linked in a common brain network ^13^, we next investigated whether the three cross-disorder factors may also share common network configurations (Fig. 4b, 55 networks in total). We found that 20 networks were predominantly associated with the general neural factor, primarily consisting of connections with the superior medial frontal (SMF) network (**Supplementary Table S10A**). Additionally, 18 networks were predominantly associated with the externalizing neural factor, mainly encompassing connections with the motor areas networks (Mot) (**Supplementary Table S10B**). Moreover, 17 networks were predominantly associated with the internalizing neural factor, primarily connected to the default mode network (DMN) (**Supplementary Table S10C**). However, at the network level, we observed indifferentiable Specificity Scores among the three cross-disorder networks (*F*_(2,54)_ = 0.18, *P* = 0.83). In summary, our findings indicated that the general and stratified factors exhibited increased neural specificity along the neuroanatomical coarse-fine gradient from the network level to the region level.

## Discussion

**Figure 5:**
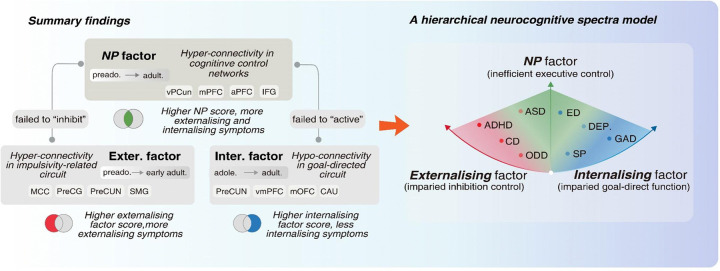
Summary of hierarchical cross-disorder neural networks.

In the present study, based on a large longitudinal cohort from adolescence to early young adulthood, we identified two stratified neural factors, respectively, underlying the externalizing and internalizing symptoms, each characterized by unique neurobiological configurations, genetic underpinnings, and clinical relevance. These two stratified neural factors, along with the previously identified general neuropsychopathological factor (*NP* factor), collectively constitute a hierarchical neurocognitive model that characterizes neural mechanisms underlying psychiatric comorbidity, with implications for early prevention and therapeutics in psychiatry (Fig. 5).

The externalizing neural factor is characterized by hyperconnectivity of primary sensory and motor regions, which has specific associations with higher impulsivity and inhibitory deficits compared to other behavioral phenotypes. This neural factor may serve as a neural mechanism underlying poor impulse control, a core dimension of externalizing psychopathology ^33,34^. Additionally, the externalizing neural factor was longitudinally predictive of externalizing symptoms across developmental stages, from preadolescence to adulthood, elucidating neural mechanisms behind the enduring impact of impulsivity on the externalizing spectrum ^35^. In contrast, the internalizing factor is characterized by hypoconnectivity of the ventromedial prefrontal cortex and medial orbitofrontal cortex, and both are crucial components of the goal-directed circuitry ^36,37^. Notably, the internalizing neural factor was specifically linked to neuroticism/negative affect traits, which predisposed individuals to experience negative emotional states and life events and was proposed as a common vulnerability factor for internalizing psychopathology ^38^. Hypoconnectivity of the internalizing neural factor might lead to challenges in responding adaptively to negative events, which perpetuate negative emotional states and result in the development of mood disorders ^39–41^.

Notably, we previously identified a general cross-disorder neural factor (*NP* factor), characterized by hyperconnectivity of executive control networks, which inefficiently regulate/support other neural networks^42^. Our studies help to clarify how the three cross-disorder networks interact in the manifestation of comorbid neuropsychopathology (summarized as a hierarchical neurocognitive spectra model in Fig. 5): externalizing comorbid symptoms may result from the combination of hyperconnectivity in the impulsivity circuit and the executive control network’s failure to inhibit impulsive behaviors, whereas internalizing comorbid symptoms may stem from the combination of the hypoconnectivity of the goal-directed circuit and the executive control network’s failure to initiate adaptive behavior.

Nevertheless, several limitations necessitate further investigation in future research endeavors. First, this study mainly focused on delineating the cross-disorder neural foundations associated with internalizing and externalizing symptoms from preadolescence to adulthood, overlooking the dimension of psychotic experiences, which typically manifest in late developmental stages ^44^. Future research could investigate whether early internalizing and externalizing neural factors (the shared and stratified ones) are risk factors for subsequent thought disorders, which, such as bipolar disorder, also demonstrate impaired behavior that falls into externalizing and internalizing domains. Second, we failed to identify a stable cross-disorder neural basis in the emotional face task, in which the standard emotional face was used to induce basic emotion. Future investigations could capitalize on more ecologically naturalistic paradigms, such as movie-watching, to illuminate the shared emotion-related neural substrates across psychiatric disorders^45^. Last, this study focused on identifying comorbidity networks at age 14 (wherein functional connections are linked to at least two psychiatric disorders at the same time). Nonetheless, previous longitudinal clinical studies had reported prevalent temporal comorbidity between psychiatric disorders ^46^, implying the presence of a transition neural network. For instance, this network was initially associated with externalizing symptoms but not internalizing symptoms, later transitioning to associate with internalizing symptoms while not externalizing symptoms. Integrating persistent and transition comorbidity networks in future research will offer a more comprehensive understanding of the evolution and interaction mechanisms underlying psychiatric comorbidity.

In conclusion, we identified two cross-disorder neural factors for internalizing and externalizing symptoms that persist longitudinally from adolescence to early adulthood. The two stratified neural factors demonstrated neuroanatomical specificity and are further delineated by cognitive, behavioral and genetic risk factors. Combining with the previously identified general *NP* factor, we present a hierarchical neurocognitive spectra model for psychiatric comorbidity. These findings might help provide a unified neurobehavioral-based psychiatric nosology that could improve diagnostic precision and treatment efficacy ^43^.

## Methods

### Study overview.

In the present study, we aimed to identify a stratified cross-disorder neural factor for externalizing and internalizing symptoms with multivariate associations between psychiatric symptoms and task-based functional connectivity, which were estimated at age 14. These multivariate associations have also been used in the identification of general neural factor in the previous study ^5^. Briefly, we employed a mutually exclusive approach to identify specific externalizing and internalizing edges. For example, externalizing edges, composed of functional connectivity, were predictive only of externalizing symptoms and not internalizing symptoms, and vice versa for internalizing edges. Only those stratified edges surviving permutation-based reliability analysis were identified as the externalizing or internalizing neural factor. Then, we checked the longitudinal persistence of the externalizing and internalizing neural factors across age 19 and age 23. Last, we conducted multilevel specificity characterisations of the externalizing and internalizing neural factors, such as anatomical, cognitive-behavioral, genetic and generalisation analyses. Detailed information about the psychiatric questionnaire (Development and Well-Being Assessment (DAWBA) and the Strengths and Difficulties Questionnaire (SDQ)), task design (Monetary incentive delay (MID) task, Stop-signal task (SST) and Emotional face task (EFT)), and brain-behaviour modelling were provided in the Supplementary Method.

### Externalizing and internalizing factor.

The stratified neural factor was constructed to represent specific brain signatures to externalizing or internalizing symptoms. Specifically, we first removed the general cross-disorder edges that were associated with both externalizing and internalizing symptoms simultaneously ^5^. Next, we identified two types of stratified cross-disorder edges: (1) the externalizing edges that predict at least two externalizing symptoms and (2) the internalizing edges that predict at least two internalizing symptoms. Then, for each task condition, we investigated if the number of stratified edges identified was significantly higher than a random observation using a permutation test (see the supplementary methods for more details). Only the significant task conditions and their stratified edges were kept in the following analyses. Next, to improve interpretability of results, the stratified edges were split into four different groups according to the predictive effect directions: positive-positive (or negative-negative) edges that have the same predictive effect to externalizing/internalizing symptoms positively (or negatively); positive-negative (or negative-positive) edges that have different predictive directions to externalizing/internalizing symptoms. We found that only the positive-positive externalizing edges (i.e. positively associated with externalizing symptoms) and the negative-negative internalizing edges (i.e. negatively associated with internalizing symptoms) were significantly higher than a random observation. Therefore, the two groups of stratified edges were termed the externalizing factor (positive-positive externalizing edges) and internalizing factor (negative-negative internalizing edges), respectively, and used in the following analyses.

### Specificity score of cross-disorder neural factors.

In delineating the specific underpinnings of each cross-disorder neural factor, we devised a specificity score for the brain measurements, assessing their relative contribution. At the brain region level, we initially normalized the region degree by dividing the total sum of degrees for each cross-disorder factor respectively, then plus 1 to prevent potential singularities in subsequent calculations. This normalized region degree ranged from 1 to 2, indicating the relative importance of each brain region to the cross-disorder neural factor. Last, for each brain region, the normalized brain degree was divided by that of the other two cross-disorder neural factors, and the sum of these two ratios is then calculated as the specificity score. This score reflects the importance of this brain region to the cross-disorder factor and controls for its influence on the other two cross-disorder factors. The same computational steps were applied at the network level.

### Generalization datasets.

To investigate whether the two cross-disorder factors identified with the adolescent population-based IMAGEN dataset could be generalized into other developmental periods and clinical conditions, we utilised multiple large-scale population-based datasets (the Adolescent Brain Cognitive Development cohort^31^, **ABCD**) and clinical case-control datasets (**The STRATIFY and ESTRA**^32^, **ADHD-200**^48^, **ABIDE II; XiNan**). The details of these datasets are provided in the supplementary method.

## Data Availability

IMAGEN data are available from a dedicated database: https://imagen2.cea.fr; Stratify data are also available from the IMAGEN database: https://imagen2.cea.fr; ABCD data are available from a dedicated database: https://abcdstudy.org/; HCP data are available from a dedicated database: https://www.humanconnectome.org/; ADHD-200 data are available from a dedicated database: http://fcon_1000.projects.nitrc.org/indi/adhd200. ABIDEII data are available from a dedicated database: http://fcon_1000.projects.nitrc.org/indi/abide/abide_II.html. XiNan and STRATIFY/ESTRA datasets are available from the principal investigator of the study and are subject to local ethics committee requirements. Shen 268 parcellation is available from https://www.nitrc.org/frs/?group_id=51.

## References

[1] Fusar-Poli P., Transdiagnostic psychiatry: a systematic review. World psychiatry: official journal of the World Psychiatric Association (WPA) 18, 192–207 (2019). 31059629 10.1002/wps.20631 PMC6502428

[2] Regier D.A., Kuhl E.A. & Kupfer D.J. The DSM-5: Classification and criteria changes. World psychiatry 12, 92–98 (2013). 23737408 10.1002/wps.20050 PMC3683251

[3] Caspi A. & Moffitt T.E. All for One and One for All: Mental Disorders in One Dimension. Am J Psychiatry 175, 831–844 (2018). 29621902 10.1176/appi.ajp.2018.17121383 PMC6120790

[4] Rodriguez-Seijas C., Diversity and the Hierarchical Taxonomy of Psychopathology (HiTOP). Nature Reviews Psychology (2023).

[5] Xie C., A shared neural basis underlying psychiatric comorbidity. Nat Med 29, 1232–1242 (2023). 37095248 10.1038/s41591-023-02317-4 PMC10202801

[6] Insel T., Research domain criteria (RDoC): toward a new classification framework for research on mental disorders. Am J Psychiatry 167, 748–751 (2010). 20595427 10.1176/appi.ajp.2010.09091379

[7] Hoy N., Lynch S.J., Waszczuk M.A., Reppermund S. & Mewton L. Transdiagnostic biomarkers of mental illness across the lifespan: A systematic review examining the genetic and neural correlates of latent transdiagnostic dimensions of psychopathology in the general population. Neurosci Biobehav Rev 155, 105431 (2023). 37898444 10.1016/j.neubiorev.2023.105431

[8] Brett M., Johnsrude I.S. & Owen A.M. The problem of functional localization in the human brain. Nature reviews neuroscience 3, 243–249 (2002). 11994756 10.1038/nrn756

[9] McTeague L.M., Identification of Common Neural Circuit Disruptions in Cognitive Control Across Psychiatric Disorders. Am J Psychiatry 174, 676–685 (2017). 28320224 10.1176/appi.ajp.2017.16040400 PMC5543416

[10] McTeague L.M., Identification of Common Neural Circuit Disruptions in Emotional Processing Across Psychiatric Disorders. Am J Psychiatry 177, 411–421 (2020). 31964160 10.1176/appi.ajp.2019.18111271 PMC7280468

[11] Seguin C., Sporns O. & Zalesky A. Brain network communication: concepts, models and applications. Nat Rev Neurosci 24, 557–574 (2023). 37438433 10.1038/s41583-023-00718-5

[12] Van Essen D.C. & Barch D.M. The human connectome in health and psychopathology. World Psychiatry 14, 154–157 (2015). 26043324 10.1002/wps.20228 PMC4471963

[13] Segal A., Regional, circuit and network heterogeneity of brain abnormalities in psychiatric disorders. Nat Neurosci 26, 1613–1629 (2023). 37580620 10.1038/s41593-023-01404-6 PMC10471501

[14] Siddiqi S.H., Brain stimulation and brain lesions converge on common causal circuits in neuropsychiatric disease. Nat Hum Behav 5, 1707–1716 (2021). 34239076 10.1038/s41562-021-01161-1 PMC8688172

[15] van den Heuvel M.P. & Sporns O. A cross-disorder connectome landscape of brain dysconnectivity. Nature Reviews Neuroscience (2019). 10.1038/s41583-019-0177-6 PMC8864539 31127193

[16] Fornito A., Zalesky A. & Breakspear M. The connectomics of brain disorders. Nat Rev Neurosci 16, 159–172 (2015). 25697159 10.1038/nrn3901

[17] Elliott M.L., Romer A., Knodt A.R. & Hariri A.R. A Connectome-wide Functional Signature of Transdiagnostic Risk for Mental Illness. Biol Psychiatry 84, 452–459 (2018). 29779670 10.1016/j.biopsych.2018.03.012 PMC6119080

[18] Romer A.L., Structural alterations within cerebellar circuitry are associated with general liability for common mental disorders. Molecular psychiatry 23, 1084–1090 (2018). 28397842 10.1038/mp.2017.57 PMC5636639

[19] Xia C.H., Linked dimensions of psychopathology and connectivity in functional brain networks. Nat Commun 9, 3003 (2018). 30068943 10.1038/s41467-018-05317-y PMC6070480

[20] Xie C., Reward Versus Nonreward Sensitivity of the Medial Versus Lateral Orbitofrontal Cortex Relates to the Severity of Depressive Symptoms. Biol Psychiatry Cogn Neurosci Neuroimaging 6, 259–269 (2021). 33221327 10.1016/j.bpsc.2020.08.017

[21] Krueger R.F. & Eaton N.R. Transdiagnostic factors of mental disorders. World Psychiatry Official Journal of the World Psychiatric Association 14, 27–29 (2015). 10.1002/wps.20175 PMC4329885 25655146

[22] Paus T., Keshavan M. & Giedd J.N. Why do many psychiatric disorders emerge during adolescence? Nat Rev Neurosci 9, 947–957 (2008). 19002191 10.1038/nrn2513 PMC2762785

[23] Guo Y., Schmitz T.W., Mur M., Ferreira C.S. & Anderson M.C. A supramodal role of the basal ganglia in memory and motor inhibition: Meta-analytic evidence. Neuropsychologia 108, 117–134 (2018). 29199109 10.1016/j.neuropsychologia.2017.11.033 PMC5759998

[24] Liu X., Hairston J., Schrier M. & Fan J. Common and distinct networks underlying reward valence and processing stages: a meta-analysis of functional neuroimaging studies. Neurosci Biobehav Rev 35, 1219–1236 (2011). 21185861 10.1016/j.neubiorev.2010.12.012 PMC3395003

[25] Chen T., Cai W., Ryali S., Supekar K. & Menon V. Distinct Global Brain Dynamics and Spatiotemporal Organization of the Salience Network. PLoS Biol 14, e1002469 (2016). 27270215 10.1371/journal.pbio.1002469 PMC4896426

[26] Uddin L.Q., Salience network-based classification and prediction of symptom severity in children with autism. JAMA Psychiatry 70, 869–879 (2013). 23803651 10.1001/jamapsychiatry.2013.104 PMC3951904

[27] Carpenter K.L.H., Sensory Over-Responsivity: An Early Risk Factor for Anxiety and Behavioral Challenges in Young Children. J Abnorm Child Psychol 47, 1075–1088 (2019). 30569253 10.1007/s10802-018-0502-y PMC6508996

[28] Pannekoek J.N., Aberrant resting-state functional connectivity in limbic and salience networks in treatment–naive clinically depressed adolescents. J Child Psychol Psychiatry 55, 1317–1327 (2014). 24828372 10.1111/jcpp.12266

[29] Chambers R.A., Taylor J.R. & Potenza M.N. Developmental neurocircuitry of motivation in adolescence: a critical period of addiction vulnerability. Am J Psychiatry 160, 1041–1052 (2003). 12777258 10.1176/appi.ajp.160.6.1041 PMC2919168

[30] Kotov R., Gamez W., Schmidt F. & Watson D. Linking “big” personality traits to anxiety, depressive, and substance use disorders: a meta-analysis. Psychological bulletin 136, 768 (2010). 20804236 10.1037/a0020327

[31] Marek S., Identifying Reproducible Individual Differences in Childhood Functional Brain Networks: An ABCD Study. Developmental Cognitive Neuroscience (2019). 10.1016/j.dcn.2019.100706 PMC6927479 31614255

[32] Quinlan E.B., Identifying biological markers for improved precision medicine in psychiatry. Mol Psychiatry 25, 243–253 (2020). 31676814 10.1038/s41380-019-0555-5 PMC6978138

[33] Beauchaine T.P., Zisner A.R. & Sauder C.L. Trait Impulsivity and the Externalizing Spectrum. Annu Rev Clin Psychol 13, 343–368 (2017). 28375718 10.1146/annurev-clinpsy-021815-093253

[34] Krueger R.F., Validity and utility of Hierarchical Taxonomy of Psychopathology (HiTOP): II. Externalizing superspectrum. World Psychiatry 20, 171–193 (2021). 34002506 10.1002/wps.20844 PMC8129870

[35] Martel M.M., Levinson C.A., Lee C.A. & Smith T.E. Impulsivity Symptoms as Core to the Developmental Externalizing Spectrum. J Abnorm Child Psychol 45, 83–90 (2017). 27017822 10.1007/s10802-016-0148-6 PMC5040618

[36] Voon V., The neurochemical substrates of habitual and goal-directed control. Transl Psychiatry 10, 84 (2020). 32127520 10.1038/s41398-020-0762-5 PMC7054261

[37] Rudebeck P.H., Saunders R.C., Prescott A.T., Chau L.S. & Murray E.A. Prefrontal mechanisms of behavioral flexibility, emotion regulation and value updating. Nat Neurosci 16, 1140–1145 (2013). 23792944 10.1038/nn.3440 PMC3733248

[38] Griffith J.W., Neuroticism as a common dimension in the internalizing disorders. Psychol Med 40, 1125–1136 (2010). 19903363 10.1017/S0033291709991449 PMC2882529

[39] Paulus D.J., Vanwoerden S., Norton P.J. & Sharp C. Emotion dysregulation, psychological inflexibility, and shame as explanatory factors between neuroticism and depression. J Affect Disord 190, 376–385 (2016). 26546773 10.1016/j.jad.2015.10.014

[40] Wenzlaff R.M. & Wegner D.M. Thought suppression. Annual Review of Psychology 51, 59–91 (2000). 10.1146/annurev.psych.51.1.59 10751965

[41] Hofmann S.G., Sawyer A.T., Fang A. & Asnaani A. Emotion dysregulation model of mood and anxiety disorders. Depression and anxiety 29, 409–416 (2012). 22430982 10.1002/da.21888

[42] Johnson M.H. Executive function and developmental disorders: the flip side of the coin. Trends Cogn Sci 16, 454–457 (2012). 22835639 10.1016/j.tics.2012.07.001

[43] Michelini G., Palumbo I.M., DeYoung C.G., Latzman R.D. & Kotov R. Linking RDoC and HiTOP: A new interface for advancing psychiatric nosology and neuroscience. Clin Psychol Rev 86, 102025 (2021). 33798996 10.1016/j.cpr.2021.102025 PMC8165014

[44] Wright A.G., The structure of psychopathology: toward an expanded quantitative empirical model. J Abnorm Psychol 122, 281–294 (2013). 23067258 10.1037/a0030133 PMC3570590

[45] Qin S. Emotion representations in context: maturation and convergence pathways. Trends Cogn Sci 27, 883–885 (2023). 37598002 10.1016/j.tics.2023.07.009

[46] Plana-Ripoll O., Exploring Comorbidity Within Mental Disorders Among a Danish National Population. JAMA Psychiatry (2019). 10.1001/jamapsychiatry.2018.3658 PMC6439836 30649197

[47] Dalley J.W. & Robbins T.W. Fractionating impulsivity: neuropsychiatric implications. Nat Rev Neurosci 18, 158–171 (2017). 10.1038/nrn.2017.8 28209979

[48] Consortium H.D. The ADHD-200 Consortium: A Model to Advance the Translational Potential of Neuroimaging in Clinical Neuroscience. Front Syst Neurosci 6, 62–62 (2012). 22973200 10.3389/fnsys.2012.00062 PMC3433679

